# Vaginal *Tritrichomonas foetus* infection in mice as an *in vivo* model for drug development against *Trichomonas vaginalis*

**DOI:** 10.1371/journal.pone.0308672

**Published:** 2024-10-01

**Authors:** Noelle M. Nieskens, Yukiko Miyamoto, Brianna M. Hurysz, Anthony J. O’Donoghue, Lars Eckmann

**Affiliations:** 1 Department of Medicine, University of California San Diego, La Jolla, California, United States of America; 2 Center for Discovery and Innovation in Parasitic Diseases, Skaggs School of Pharmacy and Pharmaceutical Sciences, University of California San Diego, La Jolla, California, United States of America; New York Medical College, UNITED STATES OF AMERICA

## Abstract

*Trichomonas vaginalis* is the causative agent of the common sexually transmitted disease, trichomoniasis, which affects more than a hundred million people worldwide. Metronidazole and tinidazole, agents belonging to the 5-nitroheterocyclic class of antimicrobials, are most often used to treat infection, but increased resistance has been reported and adverse effects of these drugs can be significant. Consequently, an urgent need exists for the development of novel drug entities against trichomoniasis. Critical for antimicrobial drug development is the demonstration of *in vivo* efficacy. Murine models of vaginal *T*. *vaginalis* infection are unreliable for unknown reasons. Meanwhile, murine infections with the related bovine pathogen, *Tritrichomonas foetus*, tend to be more robust, although susceptibility to different antimicrobials might differ from *T*. *vaginalis*. Here, we explored the utility of *T*. *foetus* infection as a surrogate model for drug development against *T*. *vaginalis*. Four different *T*. *foetus* strains caused robust vaginal infection in young mice, while none of four diverse *T*. *vaginalis* strains did. Comparison of drug susceptibility profiles revealed that *T*. *foetus* and *T*. *vaginalis* were similarly susceptible to a range of 5-nitroheterocyclic and gold(I) compounds. By comparison, proteasome inhibitors were 10- to 15-fold less active against *T*. *foetus* than *T*. *vaginalis*, although one of the proteasome inhibitors, bortezomib, had low micromolar activity or better against multiple strains of both trichomonads. Different strains of *T*. *foetus* were used to demonstrate the utility of the murine vaginal infection models for *in vivo* efficacy testing, including for bortezomib and a gold(I) compound. The differences in susceptibility to proteasome inhibitors may be partially explained by differences in the proteasome subunit sequences between the two trichomonads, although the functional relevance of the proteasome was similar in both organisms. These findings indicate that *T*. *foetus* can serve as a reliable surrogate model for *T*. *vaginalis in vitro* and in murine infections *in vivo*, but caution must be exercised for specific drug classes with targets, such as the proteasome, that may display genetic divergence between the trichomonads.

## Introduction

Trichomoniasis, caused by infection with the protozoan parasite *Trichomonas vaginalis*, is the most common, non-viral sexually transmitted disease in the world with a global incidence of ~156 million, and a global prevalence of 5.4% [1]. The parasite can infect different genitourinary tissues, including the urethra, vagina, and cervix of women, and the prostate and urethra of men [2]. Infected women can experience a broad range of symptoms, including abnormal vaginal discharge, odor, and itching, vulvar or vaginal erythema, and colpitis macularis (“strawberry cervix”) [3]. Many women remain infected for extended periods [3]. Infection in men can lead to mild to moderate urethral discharge and urethral inflammation, but symptoms tend to be less severe than in women [1, 4]. The global prevalence of *T*. *vaginalis* infection is almost 10 times lower in men compared to women [1], which may reflect a lack of testing due to milder infection symptoms or could be related to an abbreviated course of infection. Nonetheless, lack of symptoms does not protect an individual from the complications associated with infection. For example, *T*. *vaginalis* infection can increase susceptibility to human immunodeficiency virus (HIV) infection by 50% [5]. Furthermore, a positive correlation between HIV infection and trichomoniasis symptom severity was found in men [6]. In women, infection has also been linked to an increased risk of cervical cancer, low infant birth weight, preterm birth, and stillbirths [7, 8]. In men, a decrease in sperm viability and motility has been observed with the risk to decrease fertility [9]. To avoid potential complications, it is important to treat infection upon diagnosis, particularly since infection can last for months to years [10].

Metronidazole and tinidazole are two antimicrobials currently FDA-approved for the treatment of trichomoniasis [11]. They belong to the class of 5-nitroheterocyclic drugs. Metronidazole, first developed in 1959, is the primary drug for trichomoniasis treatment worldwide [12]. Tinidazole, a structural derivative of metronidazole, was FDA-approved for trichomoniasis treatment in 2004 [11]. Both are prodrugs that must be reduced to a free radical inside microbial cells before they can covalently bind to multiple protein and nucleic acid targets. Adduct formation leads to inhibition of DNA synthesis and many vital protein functions, eventually killing the organism. Both drugs primarily target anaerobic microorganisms, including *T*. *vaginalis*, as these organisms possess enzymatic pathways with low enough redox potential to donate electrons to the drug molecules [12]. For metronidazole, cure rates range from 86–97% [13–15]. Despite its general effectiveness, an estimated 2–10% of all clinical trichomoniasis cases are caused by strains that display resistance to metronidazole and other 5-nitroheterocyclic drugs [16–18]. Beyond loss of efficacy due to resistance, metronidazole can have significant adverse effects that compromise its use and effectiveness. Thus, a large percentage of women treated with the drug have reported nausea, vomiting, unpleasant taste, diarrhea, headache, vaginal irritation, and yeast infections [19, 20]. Currently no other drug classes are FDA-approved to treat trichomoniasis. With the threat of growing resistance, and significant adverse effects, development of alternative antimicrobials in other drug classes remains urgent for treatment of trichomoniasis.

Library screens of approved FDA drugs and drug candidates have been done to identify new activities against *T*. *vaginalis* but have not resulted in promising candidates [21]. *De novo* drug design against unexploited targets is currently the most realistic strategy for development of new antitrichomonal agents. One such strategy is exemplified by proteasome inhibitors [22]. The proteasome is an enzymatic complex present in all eukaryotic and some prokaryotic cells, whose function is to degrade and recycle proteins that are misfolded or not needed [23]. The enzyme complex consists of a barrel-like structure, consisting of two pairs of rings with seven α-subunits and seven β-subunits. Peptide substrates are fed through the center of the barrel where they are cleaved into smaller fragments. Three of the 14 proteasome subunits are catalytically active: β1 (caspase-like), β2 (trypsin-like), and β5 (chymotrypsin-like). Each cleaves the substrate at its preferred site via hydrolysis [24]. Proteasome inhibitors were first developed as anti-inflammatory agents and as novel agents for cancer treatment [25]. For example, the proteasome inhibitor bortezomib, was FDA-approved for the treatment of multiple myeloma and mantle cell lymphoma. More recently, proteasome inhibitors have also been found to be effective against *Plasmodium falciparum*, the protozoan responsible for malaria [26]. Importantly, several new proteasome inhibitors were shown to be highly selective for the parasite over human cells [27]. *T*. *vaginalis* also possess a proteasome that can be targeted for the development of new agents against the parasite, since several proteasome inhibitors were active against the parasite *in vitro* and have selectivity over human cells [22].

Critical for antimicrobial drug development is the demonstration of *in vivo* efficacy. Ideally, animal models of infection can be used that employ the very same pathogens that infect humans. In the case of trichomoniasis, several reports have shown that mice can be infected vaginally with *T*. *vaginalis* [28–30], but infections tend to be tenuous and unreliable for poorly understood reasons. As an alternative, mouse infection models with surrogate trichomonad pathogens can be used if it can be shown that those infections are robust, and the pathogens respond to antimicrobial candidates in similar ways to the human target pathogen. For example, vaginal infection of mice with the bovine pathogen, *Tritrichomonas foetus*, have been shown to cause sustained infection [28]. *T*. *foetus* is a protozoan parasite that causes tritrichomoniasis in cattle, a similar disease to human trichomoniasis. The parasite is closely related to *T*. *vaginalis*, being in the same phylum [31]. Despite its promise, the broader utility of this model for drug development against *T*. *vaginalis* remains to be established. Therefore, the aims of this study were to establish infectious characteristics in mice, similarities in *in vitro* drug responses, structural target similarities of a set of *T*. *foetus* strains in relation to *T*. *vaginalis* and establish the overall utility and limits of the murine *T*. *foetus* model as a basis for current and future drug development against *T*. *vaginalis*.

## Materials and methods

### Trichomonad strains

*T*. *foetus* strains 003 (ATCC 30003), 166 (ATCC 30166), 232 (ATCC 30232), 924 (ATCC 30924), and *T*. *vaginalis* strain G3 (ATCC PRA-98) were obtained from the American Type Culture Collection (Manassas, VA). *T*. *vaginalis* strains R88, MSA1172, S520 and S1489 were kindly provided by Dr. William Secor (Centers for Disease Control and Prevention, GA). *T*. *foetus* strain D1 and *T*. *vaginalis* strain F1623 were described in prior studies [32, 33]. Trichomonads were grown in trypticase-yeast extract-maltose (TYM) medium, supplemented with 10% horse serum (Omega), 0.74% ammonium iron (II) sulfate hexahydrate (Sigma-Aldrich) and 1% penicillin-streptomycin (Sigma-Aldrich), and adjusted to pH 6.2. Cultures were grown axenically at 37°C under anaerobic conditions.

### Antimicrobial compounds

The following 5-nitro heterocyclic were purchased commercially (molecular masses in parentheses): Metronidazole (171 Da; Sigma-Aldrich), ronidazole (200 Da; APExBIO), tinidazole (247 Da; TCI America), nitazoxanide (307 Da; AdipoGen), and nithiamide (187 Da; MedChemExpress). The following gold(I) compounds (CPD), whose naming follows our prior usage [34], were also purchased: Auranofin (679 Da; Enzo Life Sciences), (Tri-*n*-ethylphosphine)gold(I) chloride (351 Da, CPD4; Sigma-Aldrich), (Tri-*n*-methylphosphine)gold(I) chloride (309 Da, CPD10; Sigma-Aldrich) and myochrysine (390 Da; Sigma-Aldrich). The following gold(I) compounds were synthesized and characterized as described previously [34]: (Tri-*n*-propylphosphine)gold(I) chloride (357 Da, CPD11), (Triisopropylphosphine)gold(I) chloride (357 Da, CPD12), Bis(triethylphosphine)gold(I) chloride (433 Da, CPD14), and Bis(tri-*n*-propylphosphine)gold (I) chloride (517 Da, CPD15). The proteasome inhibitors bortezomib (384 Da), ixazomib (361 Da) and carfilzomib (720 Da) were obtained from MedChemExpress. The benzimidazole compounds lansoprazole (369 Da), omeprazole (345 Da), albendazole (281 Da) and fenbendazole (299 Da) were also obtained from MedChemExpress. Pantoprazole (383 Da) was purchased from APExBIO and disulfiram (297 Da) from TCI America.

### Mice

BALB/cJ mice (obtained from The Jackson Laboratory) were bred in-house. Female mice were used within one week of weaning at 3–6 weeks of age. Groups of mice were housed in single plastic cages with free access to feed and water.

The study was carried out in strict accordance with the recommendations in the Guide for the Care and Use of Laboratory Animals of the National Institutes of Health. The protocol was approved by the University of California San Diego Institutional Animal Care and Use Committee (Protocol Number: S00205). Mice were handled with care, only by research staff fully trained in animal handling, at all times to minimize distress. Mouse health was monitored daily, as well as each time a sample was taken. Vaginal trichomonad infections in mice cause no pain, distress, or suffering, obviating any need for humane endpoints. Experimental endpoints were based solely on the infectious dynamics relative to the study objectives of assessing drug effects on infectious load. All animals were euthanized on the last day of sample collection by controlled CO_2_ inhalation, followed by cervical dislocation.

### Vaginal infections and treatment

Infections were performed with different strains of either *T*. *vaginalis* or *T*. *foetus* as indicated under the specific experiments in the figures. Trichomonads were cultured overnight, centrifuged, and resuspended at a density of 2 x 10^8^ trophozoites/mL. For all *in vitro* and *in vivo* experiments, numbers of viable trophozoites were determined under a phase-contrast microscope. Viability was >95% as assessed by observation of the characteristic parasite motility. Five μL of the parasite suspension (containing 10^6^ trophozoites) were slowly administered intravaginally to mice with a 20 μL micropipette. Mice were held briefly upside-down after agent administration to minimize leakage.

In select experiments (as indicated in the figure legends), mice were pretreated one day before infection with one of following pretreatment regimens: a) 5 μL intravaginal administration of 10 mg/mL ammonium iron (III) citrate (Sigma-Aldrich); b) 100 μL intraperitoneal (i.p.) injection of 10 mg/mL ammonium iron (III) citrate; c) 100 μL i.p. injection of 4 mg/mL 17β-estradiol (Sigma-Aldrich); or d) 100 μL i.p. injection of 4 mg/mL 17β-estradiol (Sigma-Aldrich) and 100 μL i.p. injection of 1 mg/ml dexamethasone (Sigma-Aldrich). The ammonium iron (III) citrate solution for intravaginal administration was made in PBS containing 1% hypromellose, while all formulation for i.p. injections were prepared in PBS. Control mice received no pretreatment.

For drug efficacy tests *in vivo*, suspensions of bortezomib or gold (I) CPD4 were prepared at 10 mg/mL (= 1% w/v) in 0.2% hypromellose in PBS. Hypromellose solution without drugs was used as a control. Five μL of the drug suspensions (containing 0.05 mg of drug) were administered intravaginally as outlined above for the parasite inoculations. A total of five doses were administered: One dose on day 1 after infection, two doses each on days 2 and 3 after infection with 6–8 h between treatments on those days.

Infectious load was determined 3–4 days after infection for all experiments except for a time course study with *T*. *foetus* strain 232, where mice were sampled on days 1, 3, 6, and 9. For assessment of infection, mice were lavaged intravaginally by slowly dispensing 30 μL of TYM medium into the vagina, taking it up again, and repeating the process two more times. The washes were diluted to 120 μL in TYM medium, and 10 μL of the diluted washes were placed into a hemocytometer for counting of motile trophozoites under a phase-contrast microscope. Infectious load is expressed as total number of trophozoites per mouse. For washes without countable trophozoites, the remaining volume was cultured in 2 mL of TYM medium containing 100 units/ml penicillin and 100 μg/ml streptomycin for five days at 37°C, and regularly monitored for cell growth.

### *In vitro* drug testing

Drug stocks were prepared as 10 mM solutions in DMSO. A 75 μM working stock was prepared in PBS and 1:3 serial dilutions were made in TYM medium in 96-well plates (post-dilution volume 40 μL/well). Parasite cultures were grown overnight in a 15 mL tube, centrifuged and resuspended to a density of 7 x 10^5^–2 x 10^6^ trophozoites/mL. Ten μL of this suspension were added into each well (final culture volume 50 μL/well), resulting in a final number of 7,000–20,000 trophozoites per well and final drug concentrations from 20 μM to 1 nM. Plates were incubated at 37°C for 24 hours under anaerobic conditions (AnaeroPack; Mitsubishi Gas Chemical). Viable cell numbers were determined with a luciferase-based ATP assay (Promega) [35]. Luminescence was measured with the SpectraMax M2^e^ plate reader, using the SoftMax Pro program (version 7.0.3).

To assay the minimum lethal concentration (MLC), drugs were added to 96-well plates, and serially 2-fold diluted in TYM medium. Parasites were added to each well at a density of 7 x 10^5^/mL (*T*. *foetus* D1) or 10^6^ trophozoites/mL (*T*. *vaginalis* R88), resulting in a final number of 28,000 or 40,000 trophozoites per well, respectively. Plates were incubated at 37°C for 24 hours under anaerobic conditions. Cultures (200 μL) were then transferred to medium-filled 15 mL tubes (representing a 1:75 dilution of the overnight culture to ensure adequate dilution of the drugs in those cultures) and kept at 37°C for a maximum of five days for observation of any cell growth indicative of survivors of the initial drug exposure.

### Proteasome analysis

For sequence analysis of proteasome subunits, the genome and proteome sequences of the following parasite strains were used: *T*. *foetus* CC09-1 (GenBank AIF54491–504), *T*. *vaginalis* G3 (BioProject PRJNA938429, PRJNA16084) and *P*. *falciparum* NF54 (BioProject PRJNA422809), along with that of humans (BioProject PRJNA807723) and mice (BioProject PRJNA169). A phylogenetic tree was generated using MEGA software (version 11.0.13). Support values were calculated by bootstrapping with 100 replications. Protein sequences were aligned with the MUSCLE algorithm and an unrooted tree was constructed using the Le Gascuel model, with gamma distribution and invariant sites. Gaps were partially deleted, so that sites with missing data were only removed when necessary. Proteasome subunit designation followed the findings in [36].

For immunoblotting, *T*. *foetus* D1 and *T*. *vaginalis* F1623 cultures were incubated at 37°C for 1 hour with 0.3 μM or 3 μM of bortezomib, or with DMSO as a control. Lysates were made with radioimmunoprecipitation (RIPA) buffer (Cell Signaling Technology), supplemented with Halt protease inhibitor cocktail (Thermo Scientific). Proteins were separated on a 4–20% SDS-PAGE gel (Bio-Rad), transferred onto a PVDF membrane (Millipore) and blocked with 5% nonfat dry milk. The membrane was incubated with a primary rabbit anti-ubiquitin polyclonal antibody (Proteintech, Cat No. 10201-2-AP) at a 1:10,000 dilution overnight and a secondary horseradish peroxidase (HRP)-linked anti-rabbit IgG antibody (Cell Signaling Technology, Cat No. 7074S) at a 1:20,000 dilution for 30 minutes. Proteins were detected with a chemiluminescent HRP substrate (Millipore).

For labeling of catalytically active proteasome subunits with an activity-based probe, total cell lysates of *T*. *foetus* D1 or *T*. *vaginalis* F1623 were incubated at 37°C for 1 hour with 0.1–10 μM of bortezomib, or with DMSO as a control. Afterwards, 2 μM of the fluorescent proteasome probe, MV151 (R&D Systems, Cat No. I-190) [37] was added and lysates were further incubated at 37°C for 2 hours. Proteins were separated on a 4–20% SDS-PAGE gel, and bands were visualized by fluorescent imaging with excitation at 470 nm and emission at 530 nm.

### Statistics

All statistical analyses were performed with GraphPad Prism (version 10.0.0). For compound activity screening, luminescence values were normalized against drug-free controls and plotted against the log drug concentration in each well to create a concentration-response curve for each drug. The half-maximal inhibitory concentration (IC50), or concentration of drug needed to inhibit trophozoite growth and viability by 50%, and negative log10 of the IC50 (pIC50) were calculated. The assay sensitivity for pIC50 was set at 4.7, equivalent to the highest drug concentration tested in the assays. Each experiment was independently repeated three times per compound and per trichomonad strain.

For *in vitro* data comparisons, parametric tests (ANOVA, t-tests) were used. For comparison of multiple groups, a one-way ANOVA was performed, and if a significant difference was found, Tukey’s multiple comparisons test was applied to specific pairs. For *in vivo* data comparisons, a non-parametric Mann-Whitney test was used.

## Results

### Murine vaginal infection model with diverse strains of *T*. *foetus* and *T*. *vaginalis*

**Fig 1:**
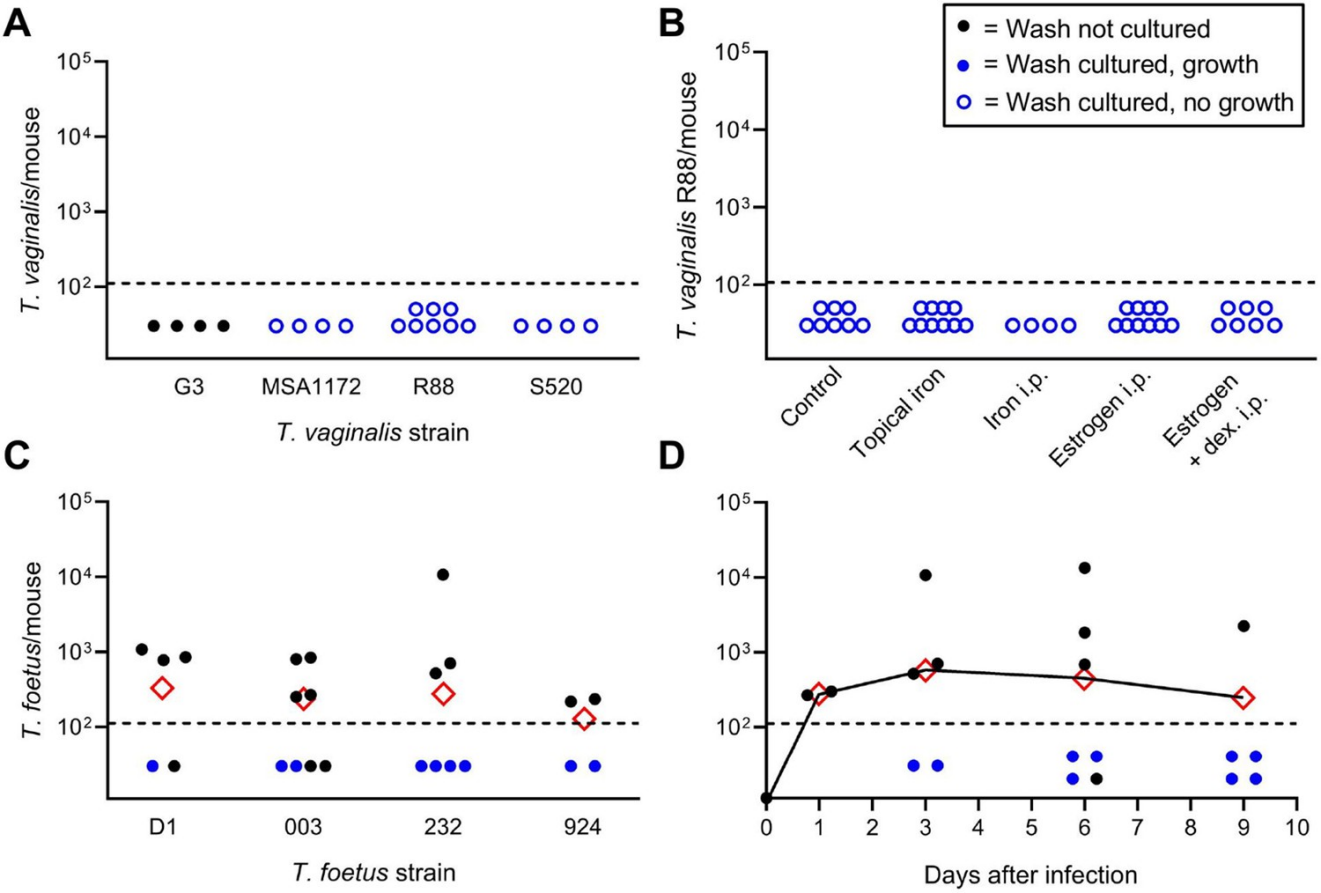
Murine susceptibility to vaginal *T*. *vaginalis* and *T*. *foetus* infection. Young (3–4 weeks) female BALB/cJ mice were intravaginally inoculated with the indicated strains of *T*. *vaginalis* (A, B) or *T*. *foetus* (C, D). In the experiments in panel B, mice were pretreated by vaginal instillation (topical) of iron or intraperitoneal (i.p.) injection of iron, estrogen, or estrogen and dexamethasone (dex) before infection, at dosages detailed in the Methods. Vaginal lavages were taken on day 3 or 4 of infection (A-C) or on the indicated days (D), and motile trophozoites were counted in a hemocytometer. Data points represent counts from individual mice, dashed lines indicate the detection threshold of the assays, and red diamonds show geometric means. For select samples without countable trophozoites, lavages were cultured in trichomonad growth medium with antibiotics (blue symbols). Positive cultures are indicated by solid blue dots, while negative cultures (no growth) are depicted by open blue dots.

Previous studies had suggested that murine vaginal infections with *T*. *vaginalis* are characterized by low infectivity and/or short-lived infections, even with various manipulations of the host designed to increase susceptibility [28]. It is possible that marginal infectivity is related to the particular strains of *T*. *vaginalis* used for the infections, so we tested a range of four diverse strains of *T*. *vaginalis* for their ability to cause vaginal infection in young female mice. Three to four days after intravaginal inoculation, vaginal washes showed no detectable trophozoite counts for any of the tested strains (Fig 1A). Cultures of the washes for up to 5 days were uniformly negative, underlining that none of the *T*. *vaginalis* strains led to even low-level infection (Fig 1A). Furthermore, systemic (i.p.) or topical (vaginal) administration of iron, which improves *T*. *vaginalis* growth in culture [38], or systemic administration of estrogen alone or estrogen plus dexamethasone, which have been suggested to improve susceptibility of mice to *T*. *vaginalis* infection [28, 39], did not promote infectivity (Fig 1B). These experiments suggest that *T*. *vaginalis* could not infect mice under any of our current conditions, regardless of particular strains or conditioning regimens.

As an alternative to *T*. *vaginalis*, we explored the related trichomonad, *T*. *foetus*, which was originally derived from cows and has been used for mouse infections [22, 28, 40]. Four different *T*. *foetus* strains were obtained and tested for their vaginal infectivity in young female mice. All four strains were able to cause infection without any pre-treatment with infectivity close to 100% (i.e., the percentage of mice with trophozoites detectable by direct counting or culture over total number of inoculated mice) (Fig 1C). To examine the time course of infection, we selected the *T*. *foetus* strain 232, which had the highest infectivity and/or highest trophozoite loads, and sampled mice every 2–3 days. All mice remained infected throughout the 9 days of the experiment, indicating a lack of spontaneous clearance during that period (Fig 1D). These findings demonstrate that *T*. *foetus* constitutes a more reliable murine model of vaginal trichomonad infection than *T*. *vaginalis*.

### Differential growth characteristics of *T*. *foetus* and *T*. *vaginalis*

**Fig 2:**
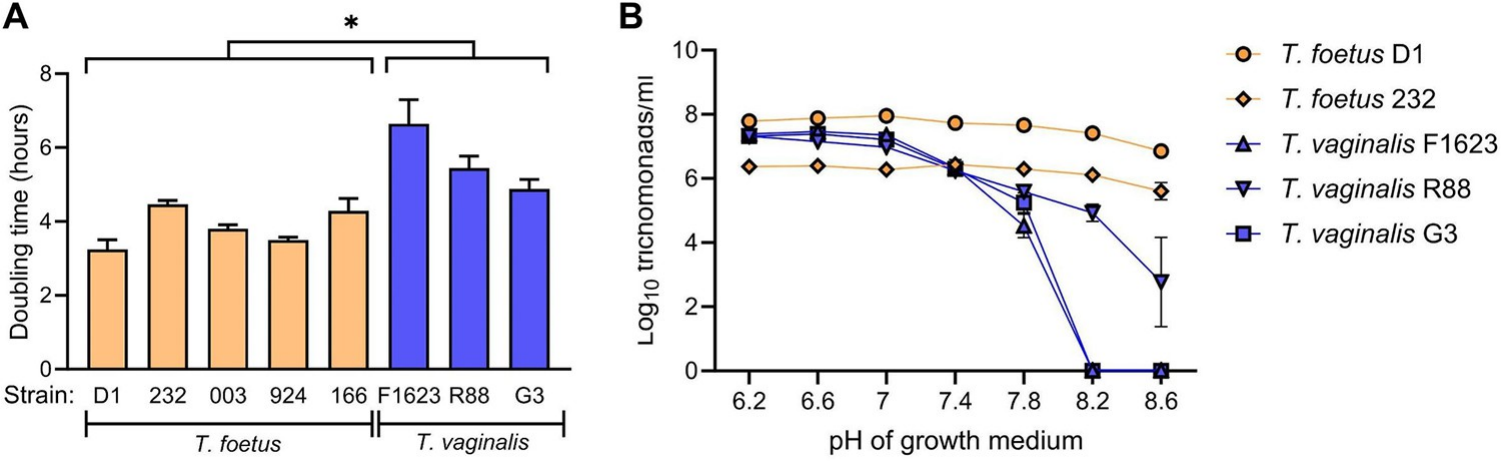
*In vitro* growth characteristics of *T*. *foetus* and *T*. *vaginalis* strains. Trophozoites of the indicated strains were cultured *in vitro* in trypticase-yeast extract-maltose (TYM) medium. (A) Cell numbers were counted before and after a 24-hour time incubation period and doubling times were calculated. Data are mean ± SE; *p<0.05 (unpaired t-test for all *T*. *vaginalis* strains vs. all *T*. *foetus* strains). (B) Media were adjusted to the indicated pH and trophozoites were grown in each medium for 24 hours and counted. Data are mean ± SE.

To explore potential reasons for the differential murine vaginal infectivity of *T*. *vaginalis* and *T*. *foetus*, we examined two parasite characteristics that may plausibly be involved: maximal growth rate and tolerance to different pH environments. The *T*. *vaginalis* strains had significantly slower growth rates in culture with longer doubling times compared to the *T*. *foetus* strains despite optimal growth conditions and media (Fig 2A). Furthermore, *T*. *vaginalis* growth was inhibited by even modestly alkaline pH *in vitro*, while *T*. *foetus* was able to grow mostly unhindered under these conditions (Fig 2B). These data indicate that the intrinsic properties of the two trichomonad species exhibit significant differences in regard to growth and pH tolerance.

To test whether the differential pH sensitivity of the trichomonad strains might be responsible for their differential infectivity in vivo, we measured the vaginal pH in 3–6 week old mice before and after treatment with estrogen alone or with estrogen and dexamethasone [28]. Mean vaginal pH was 6.21 ± 0.04 in the untreated group (mean ± SE, n = 7 mice), 6.49 ± 0.09 for the estrogen-treated group (n = 7), and 6.71 ± 0.05 for the mice treated with estrogen and dexamethasone (n = 9). A Kruskal-Wallis and Dunn’s multiple comparisons test showed significance between the estrogen and dexamethasone versus the no treatment group. However, these data show that the vaginal pH was below 7.0 in all groups, thereby excluding differential pH tolerance as a possible explanation for the differential murine vaginal infectivity of *T*. *vaginalis* vs *T*. *foetus*.

### Activity profiles of different antimicrobial drug classes against diverse *T*. *foetus* and *T*. *vaginalis* strains

To compare susceptibility to antimicrobial drugs between the two trichomonad species, we screened different strains against four major drug classes that are clinically used in trichomoniasis or have been shown to have significant *in vitro* activity against *T*. *vaginalis*: 5-nitroheterocyclic drugs [41], gold(I) compounds [34], proteasome inhibitors [22], and benzimidazoles [21]. The most consistently active compounds were 5-nitroheterocyclic compounds and gold(I) compounds (S1 Table). By comparison, the tested proteasome inhibitors had excellent activity against the *T*. *vaginalis* strains but were significantly less potent against the *T*. *foetus* strains (S1 Table). The benzimidazoles and another compound with reported *in vitro* activity against *T*. *vaginalis*, disulfiram [21], had only minimal or no activity against either *T*. *foetus* or *T*. *vaginalis* (S1 Table).

### Comparison of drug susceptibilities of *T*. *foetus* and *T*. *vaginalis*

**Fig 3:**
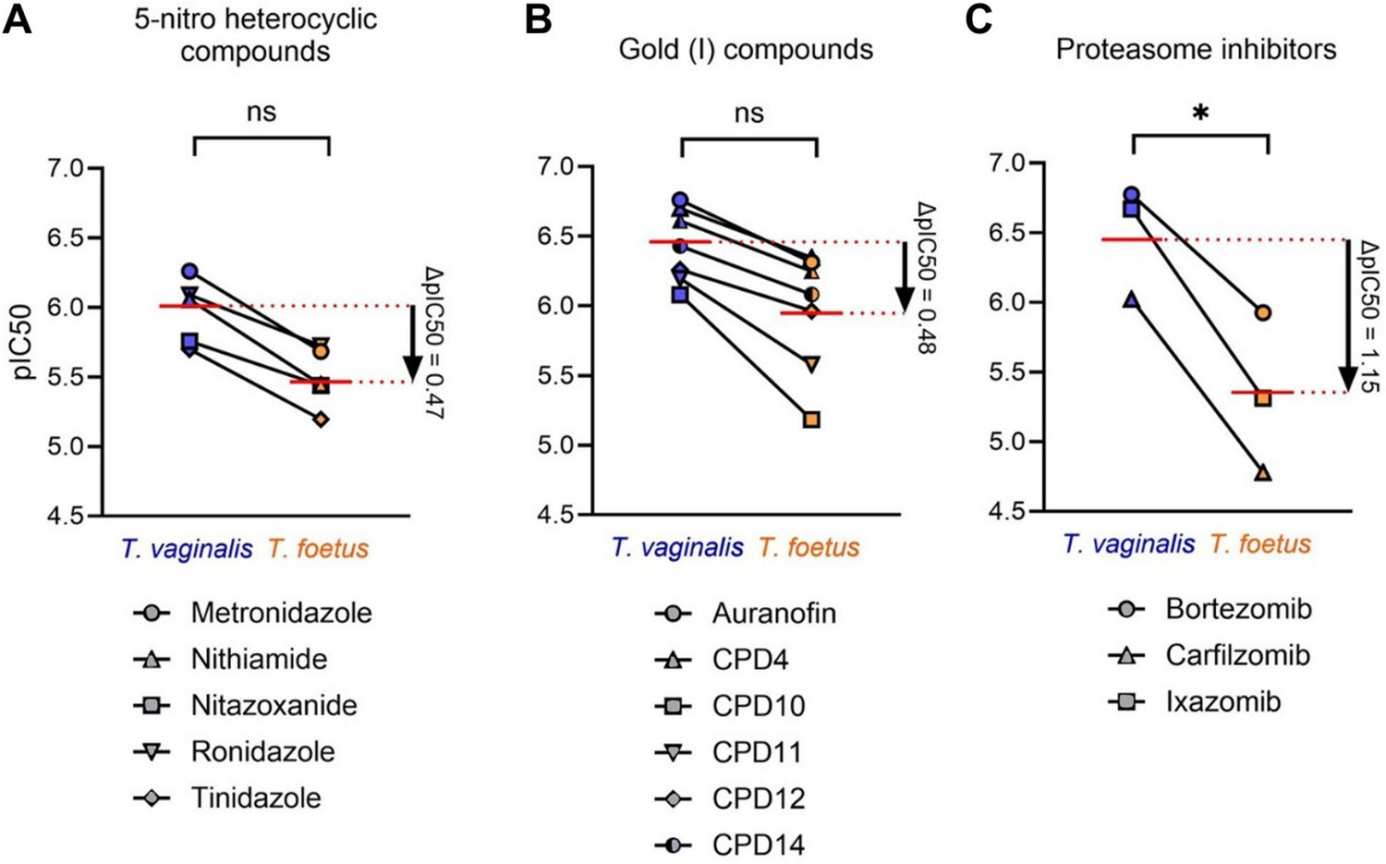
Comparison of *T*. *foetus* and *T*. *vaginalis* susceptibility to antimicrobial drugs of different classes. Activity (pIC50) of the indicated compounds from each of the indicated three drug classes against multiple strains of *T*. *foetus* and *T*. *vaginalis* was determined by 24 h growth and survival assay (the detailed data are provided in S1 Table). Each point represents the mean pIC50 of four (*T*. *foetus*) or three *(T*. *vaginalis*) different strains for the drugs listed below each graph. ΔpIC50 was calculated for each drug class by subtracting the mean *T*. *foetus* pIC50s from the mean *T*. *vaginalis* pIC50s. Red lines represent geometric means. Significance was calculated with Tukey’s multiple comparisons test (*p <0.01).

Comparison of specific drugs within the three major drug classes with the greatest potencies revealed that all tested drugs were less active in *T*. *foetus*, although differences were observed between the drug classes (Fig 3). 5-Nitroheterocyclic drugs and gold(I) drugs displayed, on average, a ~3-fold lower activity (mean ΔpIC50 = 0.47–0.48) in the *T*. *foetus* strains compared to the *T*. *vaginalis* strains, but this difference did not reach statistical significance (Fig 3). By comparison, the tested proteasome inhibitors were ~14-fold less active (mean ΔpIC50 = 1.15) against *T*. *foetus* relative to *T*. *vaginalis*, which was statistically significant (Fig 3).

**Fig 4:**
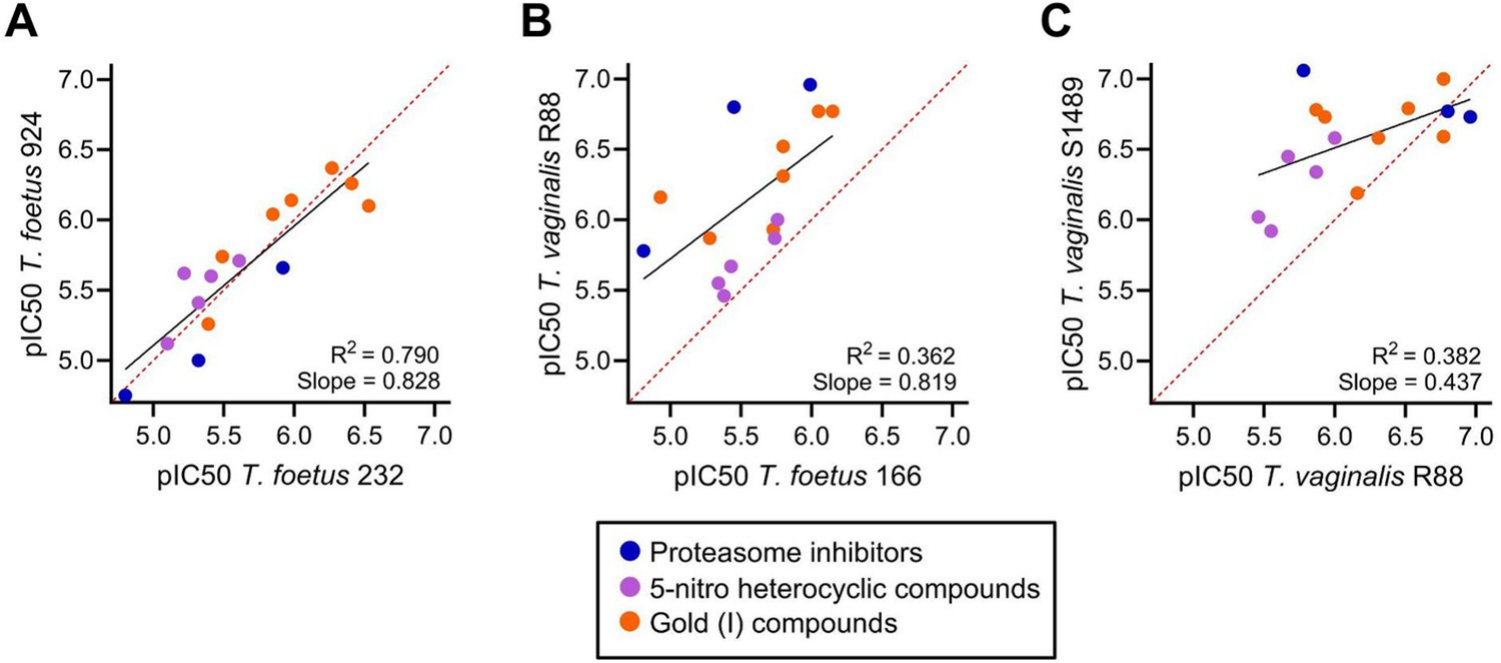
Correlation of drug susceptibility between different *T*. *foetus* and *T*. *vaginalis* strains. The graphs show correlations of the pIC50 values for individual drugs between the indicated pairs of trichomonad strains (the detailed data on drug activities used for these analyses are provided in S1 Table). Each point represents one drug and is color-coded by drug class. Plots are shown with a linear regression line (black) and line of identity (dotted red).

Pairwise comparisons of strains were conducted to discover whether any individual *T*. *foetus* strain had similar drug susceptibility profiles to any of the *T*. *vaginalis* strains. Strong positive correlations were seen among the different *T*. *foetus* strains for all three major drug classes (Fig 4A, Table 1). In contrast, only modest average correlations were observed between strains of *T*. *foetus* vs. *T*. *vaginalis* (Table 1), which particularly affected the proteasome inhibitors and gold(I) compounds (Fig 4B). This might be partially explained by the only modest average correlations of drug susceptibilities among the different *T*. *vaginalis* strains (Table 1). In particular, the *T*. *vaginalis* strain S1489 displayed a very different drug susceptibility profile than the other *T*. *vaginalis* strains (Fig 4C, Table 1), thereby markedly skewing the correlation data for both *T*. *vaginalis* and *T*. *foetus* (Table 1).

**Table 1:** Comparisons of drug responses in different *T*. *foetus* and *T*. *vaginalis* strains.

Comparisons	Strains	R^2^	Slope	P-value
*T*. *foetus* vs. *T*. *foetus*	D1 vs. 166	0.85	0.63	**0.00000046**
	D1 vs. 232	0.87	0.85	**0.00000012**
	D1 vs. 924	0.89	0.80	**0.00000003**
	166 vs. 232	0.69	1.1	**0.00006563**
	166 vs. 924	0.65	0.99	**0.00016387**
	232 vs. 924	0.79	0.83	**0.00000414**
	**Mean:**	**0.79**		
*T*. *foetus* vs. *T*. *vaginalis*	D1 vs. F1623	0.73	0.63	**0.00002580**
	D1 vs. R88	0.34	0.55	0.0172
	D1 vs. S1489	0.12	0.23	0.1819
	166 vs. F1623	0.75	0.93	**0.00001273**
	166 vs. R88	0.36	0.82	0.0137
	166 vs. S1489	0.06	0.24	0.3476
	232 vs. F1623	0.73	0.69	**0.00002257**
	232 vs. R88	0.52	0.75	**0.00153450**
	232 vs. S1489	0.21	0.33	0.0778
	924 vs. F1623	0.52	0.63	**0.00163660**
	924 vs. R88	0.17	0.45	0.1146
	924 vs. S1489	0.05	0.18	0.3939
	**Mean:**	**0.38**		
*T*. *vaginalis* vs. *T*. *vaginalis*	F1623 vs. R88	0.69	1.1	**0.00007125**
	F1623 vs. S1489	0.12	0.31	0.1886
	R88 vs. S1489	0.38	0.44	0.0107
	**Mean:**	**0.40**		

Correlation plots were generated from the pIC50 values (shown in S1 Table) of the listed pairs of trichomonad strains. A linear regression was performed on each pair to find R^2^ values. P-values state whether the slopes of the lines of best fit are significantly non-zero. Examples of graphical representations are shown in Fig 4. P-values <0.01 are bolded.

Together, these data indicate that *T*. *foetus* is generally less susceptible than *T*. *vaginalis* to all tested drugs, but this difference is particularly prominent and significant for the class of proteasome inhibitors.

### MLCs of selected drugs against *T*. *foetus* and *T*. *vaginalis*

To further characterize antimicrobial drug activity in the two trichomonad species, we determined MLCs by culturing cells for 24 hours in the presence of titrated drug concentrations, diluting the cultures 75-fold (to minimize the impact of residual drugs), and testing for survivors by outgrowth over the ensuing 5 days. The MLCs for metronidazole as a representative 5-nitroheterocyclic compound, and auranofin as a representative gold(I) compound, were very similar for *T*. *foetus* or *T*. *vaginalis*, reflecting the trends of the respective IC50 findings (Table 2). MLCs were generally 3- to 10-fold higher than IC50, consistent with the notion that complete parasite killing requires higher drug concentrations than growth inhibition by 50%. In contrast, MLC values could not be determined for the two tested proteasome inhibitors, even when tests were done with >100-fold higher drug concentrations than IC50 (Table 2). Even higher drug concentrations could not be tested in the MLC assays due to toxicity of the solvent DMSO. These results confirm that the antimicrobial activities of 5-nitroheterocyclic and gold(I) compounds are not significantly different between the two trichomonad species. The data also show that the tested proteasome inhibitors are unlikely to be trichomonacidal for either *T*. *foetus* or *T*. *vaginalis*.

**Table 2:** MLC assays of *T*. *foetus* and *T*. *vaginalis*.

				Final drug concentration (μM)				
Compound	Strain	IC50 (μM)	Experiment	Growth (+/—)				
**Metronidazole**				**0.4**	**0.8**	**1.6**	**3.2**	**6.4**
	*T*. *foetus* D1	1.5	1	+	+	+	—	—
			2	+	+	+	+	—
			3	+	+	+	—	—
	*T*. *vaginalis* R88	1.0	1	+	+	+	—	—
			2	+	+	+	—	—
			3	+	+	+	—	—
**Auranofin**				**0.4**	**0.8**	**1.6**	**3.2**	**6.4**
	*T*. *foetus* D1	0.27	1	+	+	—	—	—
			2	+	+	+	—	—
			3	+	+	+	—	—
	*T*. *vaginalis* R88	0.17	1	+	+	+	—	—
			2	+	+	+	—	—
			3	+	+	+	—	—
**Bortezomib**				**12.5**	**25**	**50**	**100**	**200**
	*T*. *foetus* D1	0.74	1	+	+	+	+	+
			2	+	+	+	+	+
			3	+	+	+	+	+
	*T*. *vaginalis* R88	0.11	1	+	+	+	+	+
			2	+	+	+	+	+
			3	+	+	+	+	+
**Carfilzomib**					**25**	**50**	**100**	**200**
	*T*. *foetus* D1	17	1		+	+	+	+
			2		+	+	+	+
			3		+	+	+	+
	*T*. *vaginalis* R88	1.7	1		+	+	+	+
			2		+	+	+	+
			3		+	+	+	+

Cultures with either *T*. *foetus* or *T*. *vaginalis* were incubated for 24 hours with indicated drugs at the listed final concentrations, diluted in media without drugs, and observed for growth for up to 5 days (+, growth; ー, no growth). Data from three independent experiments are shown. IC50 values, shown for comparison, are taken from S1 Table.

### *In vivo* efficacy testing of drugs in murine *T*. *foetus* infection models

**Fig 5:**
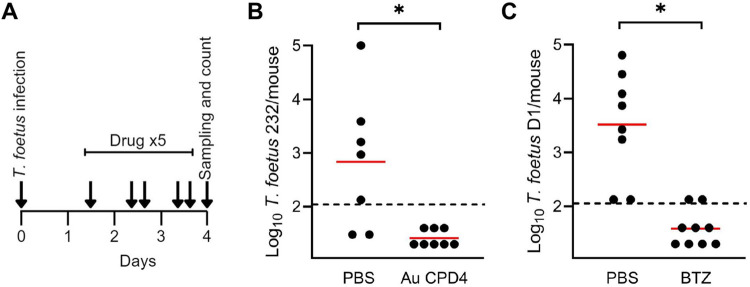
*In vivo* efficacy of selected antimicrobial compounds against *T*. *foetus*. (A) Timeline of infection and treatment. Mice were treated five times over the course of 2.5 days, starting pno day after infection. (B/C) Mice were infected with the indicated *T*. *foetus* strains and treated intravaginally with 0.05 mg/dose (5 μL of a 10 mg/mL suspension) of either the gold(I) compound CPD4 (B) or bortezomib (C), or were left untreated as controls. After 4 days, trophozoite numbers were determined. Each point represents an individual mouse. Dashed black lines represent the detection threshold. Red lines represent geometric means. Significance was calculated by Mann-Whitney test (*p <0.01).

To determine the utility of the new *T*. *foetus* vaginal infection models for drug efficacy testing, and to evaluate whether selected drugs are efficacious *in vivo*, we tested two representative compounds with excellent *in vitro* activity, the gold(I) compound, CPD4, and the proteasome inhibitor, bortezomib, using two different *T*. *foetus* strains. Mice infected with *T*. *foetus* 232 were topically treated five times over three days with CPD4 and the infectious load was determined on day 4 (Fig 5A). None of the CPD4-treated mice had countable trophozoites, while ~70% of control mice were infected with an average of ~10^3^ trophozoites per mouse (Fig 5B). Similarly, mice infected with the *T*. *foetus* strain D1 and treated with bortezomib showed no countable trophozoites, while all of the control mice had detectable infection (Fig 5C). These results demonstrate that the murine vaginal infection models are not only effective with different *T*. *foetus* strains, but are suitable for efficacy testing of new drug candidates against trichomonads. The results also reinforce the fact that CPD4 and bortezomib can clear *T*. *foetus* infection in an *in vivo* model.

### Characterization of *T*. *foetus* 20S proteasome as a drug target

**Fig 6:**
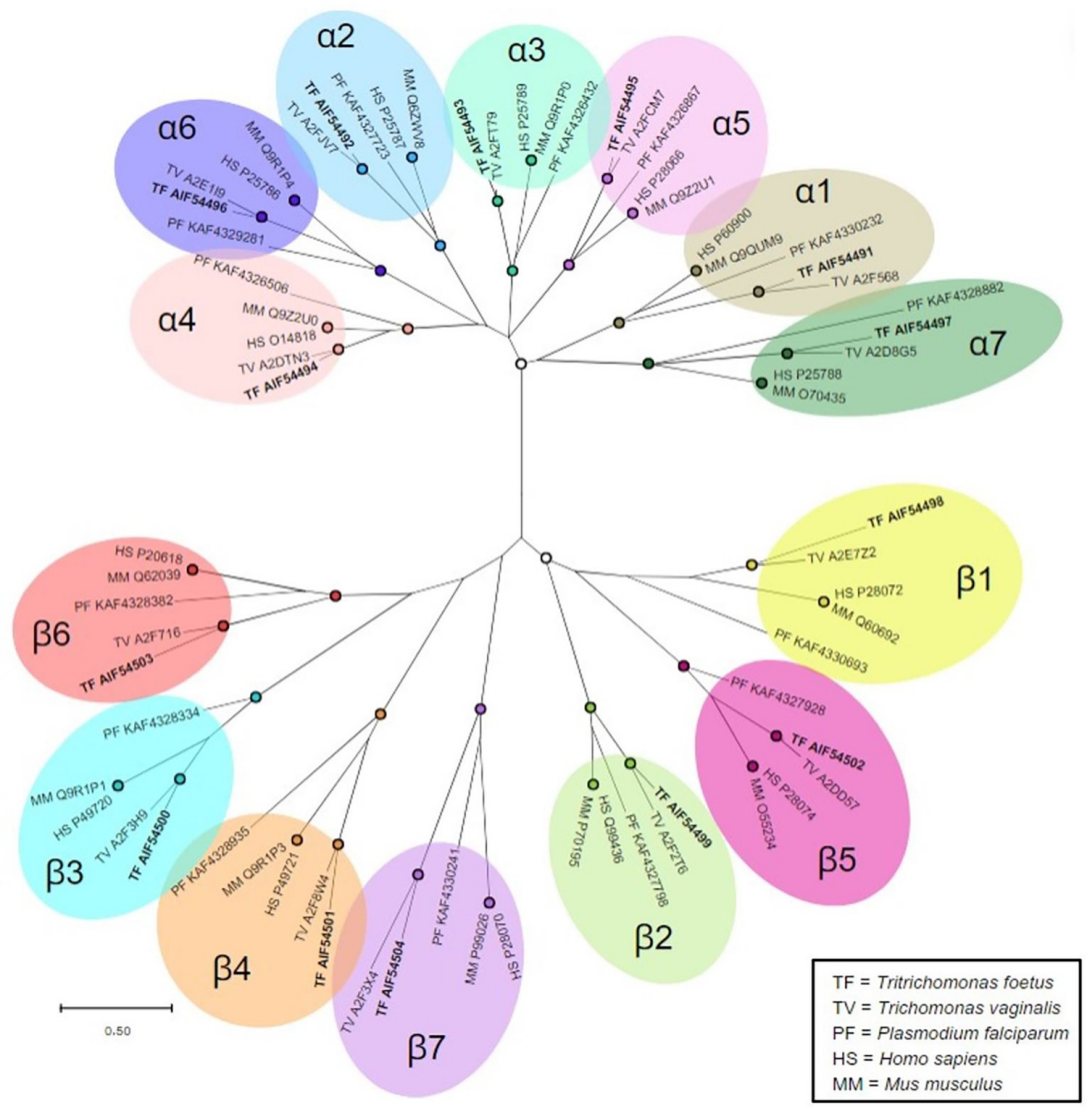
Phylogenetic analysis of trichomonad 20S proteasome subunits. Phylogenetic analysis of 20S proteasome α- and β-subunits from *T*. *foetus* (TF, bolded), *T*. *vaginalis* (TV), *Plasmodium falciparum* (PF), *Mus musculus* (MM), and *Homo sapiens* (HS). Branches are labeled with UniProt accession numbers when available, or otherwise GenBank accession numbers. Subunit groups are highlighted with the same color. Bootstrap values were calculated with 100 replications. Nodes supported with values ≥95% are marked with color-coded circles. Distance scale is shown on the bottom left.

Bortezomib showed good *in vitro* and *in vivo* activity against *T*. *foetus*, yet it and the other proteasome inhibitors were markedly less potent against the different *T*. *foetus* strains than *T*. *vaginalis* (S1 Table). This discrepancy raised the question whether the *T*. *foetus* proteasome is substantially different from that of *T*. *vaginalis*. Analysis of the *T*. *foetus* genome identified all 14 proteasome subunits, seven α subunits (α1–7) and seven β subunits (β1–7), as expected for all eukaryotes (Fig 6). Sequence comparison of the subunits showed good homology (50–87% amino acid identity) with *T*. *vaginalis*, but much less homology (31–57%) with the human and murine counterparts, or even the protozoan parasite *P*. *falciparum* (Fig 6). By comparison, the human and murine proteasome subunits are more closely related to each other than the *T*. *foetus* subunits are to *T*. *vaginalis*. These findings indicate that structural differences exist between the *T*. *foetus* and *T*. *vaginalis* proteasome subunits, potentially offering an explanation for the differential activity of the tested proteasome inhibitors against the two trichomonad species.

**Fig 7:**
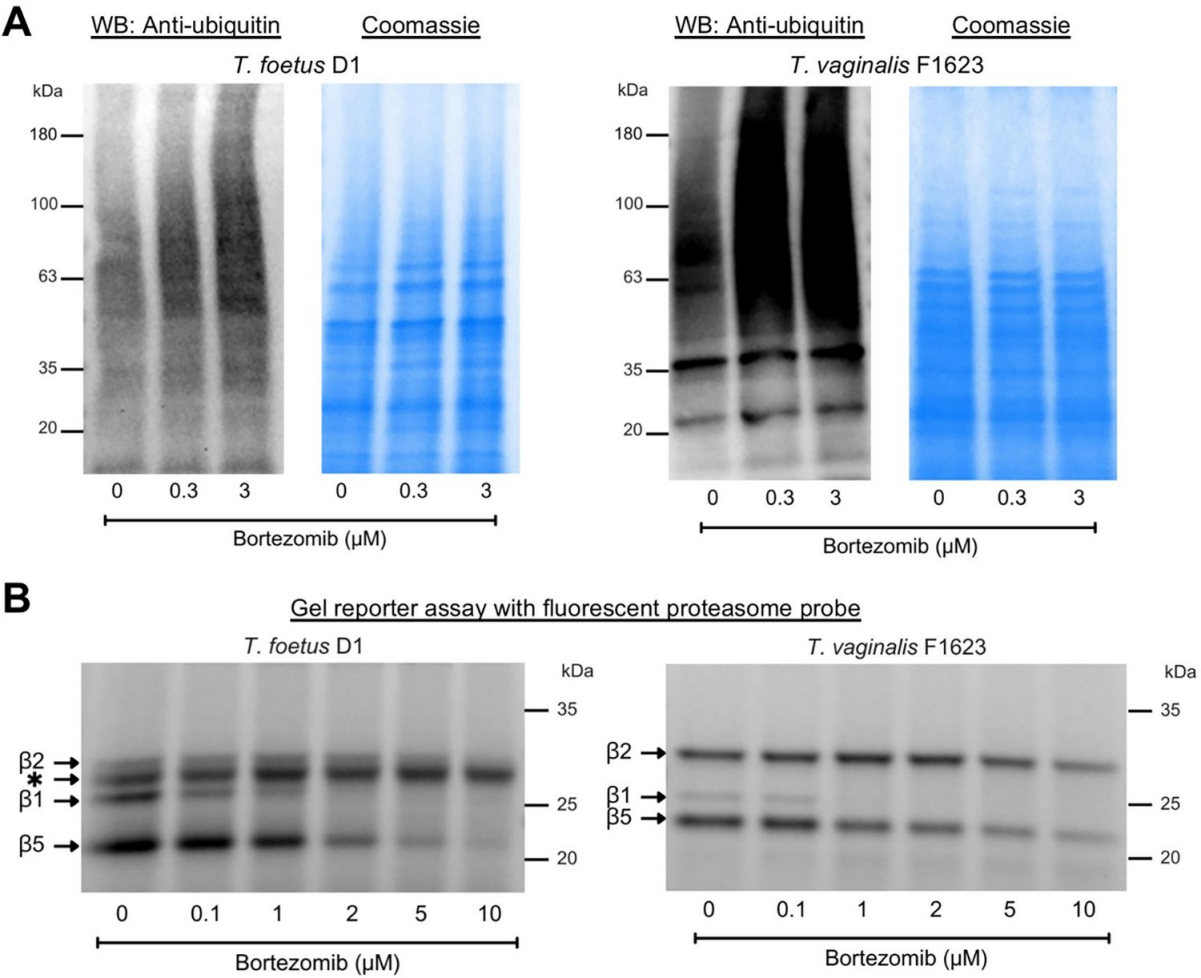
Functional analysis of trichomonad proteasomes. (A) The depicted trichomonad strains were incubated for 1 hour with the indicated final concentrations of bortezomib or left without inhibitor (0 μM). Total cell lysates were prepared and analyzed by western blotting with an anti-ubiquitin antibody (left panels). As expected, multiple proteins of different sizes are labeled with ubiquitin, leading to the apparent “smear” on the blots. Total protein was visualized by Coomassie Blue staining (right panels). (B) Total cell lysates of *T*. *foetus* and *T*. *vaginalis* were incubated for 1 hour with the indicated concentrations of bortezomib and subsequently labeled for 2 hour with the fluorescent activity-based proteasome probe, MV151. Lysates were fractionated by SDS-PAGE and labeled proteins were visualized by fluorescent imaging. The band labeled with an asterisk corresponds to a non-specific target of MV151 that could not be competed for with any available proteasome inhibitor and is thus unlikely to be a proteasome subunit. Raw images of the blots and gels are shown in S1 Raw images.

To confirm that the *T*. *foetus* proteasome is functionally similar to *T*. *vaginalis*, we analyzed the accumulation of ubiquitin-labeled proteins after proteasome inhibition by western blotting. For both trichomonads, bortezomib caused concentration-dependent increases in the abundance of ubiquitin-labeled proteins (Fig 7A). Furthermore, we used an activity-based probe, MV151 [37] for labeling of the catalytic proteasome subunits of the parasites. For *T*. *foetus*, labeling of β1 and β5 was strongly inhibited by bortezomib, while β2 labeling was only minimally affected at the highest concentrations (Fig 7B). By comparison, for *T*. *vaginalis*, inhibition was most prominent for the β1 subunit followed by β5 and, to a lesser degree, β2 (Fig 7B). Together, these findings show that bortezomib inhibits the proteasome in both *T*. *foetus* and *T*. *vaginalis*, most likely by targeting the same catalytic subunits.

## Discussion

Development of new antimicrobial drugs depends heavily on the use of suitable animal models for *in vivo* efficacy testing [40]. Robust infections with the relevant pathogens for a sufficiently long duration are key features of a good model. For trichomoniasis, infection of amenable animals, particularly rodents such as mice, with the causative pathogen, *T*. *vaginalis*, would be desirable, but we found here that murine vaginal infections with any of four *T*. *vaginalis* strains did not lead to detectable infection. In contrast, different strains of another trichomonad, *T*. *foetus*, were found to reliably infect mice for at least one week. These findings indicate that vaginal *T*. *foetus* infections provide a robust murine model suitable for *in vivo* drug testing.

The striking difference in infectivity between *T*. *vaginalis* and *T*. *foetus* is unlikely to be related to particular strains of the respective pathogen, since the findings were consistent for four diverse strains of each species. General characteristics of the two trichomonad species are probably more important. For example, we found a difference in doubling time and pH tolerance between the two trichomonad species. Faster growth rates of *T*. *foetus* might promote infectivity by counteracting inevitable vaginal losses, while the slower growth of *T*. *vaginalis* may not be sufficient to make up for such losses. Consistent with this notion, highly infectious organisms tend to have faster doubling times due to optimization of codon usage bias and increased copies of rRNA and tRNA [42]. Another potential explanation for differential infectivity could be differences in pH tolerance. The murine vagina, unlike the normally acidic environment of the human vagina, has a pH close to neutral or even slightly alkaline [43]. The ability of *T*. *foetus* to grow under alkaline pH conditions may promote vaginal infection, while *in vitro* growth of *T*. *vaginalis* was strongly inhibited by alkaline pH and may thus also be compromised *in vivo* in mice, although our measurements that the murine vaginal pH ranged from 6.5–7 would not suggest that differential pH tolerance can explain differential murine vaginal infectivity of *T*. *foetus* vs *T*. *vaginalis*.

Other possible explanations are conceivable for differential infectivity. For example, the estrus cycle of mice is characterized by periodic influx of leukocytes [44]. *T*. *foetus* may be more resistant to killing by polymorphonuclear leukocytes compared to *T*. *vaginalis* [28]. While the presence of vaginal leukocytes has no apparent impact on infectivity for *T*. *foetus*, it has been shown to have a negative impact on *T*. *vaginalis* infection [28]. Furthermore, the vaginal microbiota may be differentially affected by trichomonad infection and may impact the interactions of different trichomonads with the host. For example, *T*. *vaginalis* infection reduces vaginal epithelia-associated *Lactobacillus* species in humans [45]. In cats, *T*. *foetus* infection significantly impacts the abundance of a variety of different phyla present in the fecal microbiota [46]. Perhaps the extent and manner of microbial changes and their impact on host susceptibility to infection in mice may be more conducive to vaginal *T*. *foetus* infection.

Other studies had some success with vaginal infection of mice with *T*. *vaginalis*, although rates of infection were generally low and variable [28–30]. Beyond specific mechanistic explanations for this variable infectivity, it is conceivable that differential infectivity may be related to the host ranges of the two trichomonads. *T*. *vaginalis* is strictly a human parasite [47], while *T*. *foetus* can infect cows, cats, and pigs [48]. The apparently narrow host range of *T*. *vaginalis* may indicate a requirement for particular host features that are not present in other species, while the broader host range of *T*. *foetus* may indicate that infection depends less on particular host characteristics. It is also important to note that none of the prior work, contrary to our present study, had assessed infection by direct trichomonad counts but relied on cultures of vaginal washes. It is possible that cell numbers were too low for counting but sufficient for obtaining positive cultures, which are likely to be one or two logs more sensitive than counts. It is also possible that different conditioning or pretreatment protocols, such as multiple subcutaneous estradiol injections, or co-infection with lactobacilli could have sensitized mice to vaginal *T*. *vaginalis* infection beyond what we employed in the current work [28–30]. Finally, although we tested four different stains, differences in *T*. *vaginalis* strains could still matter for murine infectivity, so it remains possible that a heretofore untested strain can, in fact, infect mice robustly.

For a microorganism to be a useful surrogate model for drug development against a related but different infectious agent, both must have similar responses to the drugs of interest. We observed such congruency in the drug susceptibility of *T*. *foetus* and *T*. *vaginalis* to 5-nitroheterocyclic drugs and gold(I) compounds. Both drug classes are thought to act on a broad range of targets [49, 50]. In the case of 5-nitroheterocyclic drugs, the prodrug forms are first reductively activated, then form covalent adducts on multiple target molecules, including different proteins as well as nucleic acids [51]. The molecular targets responsible for cytocidal activity of 5-nitroheterocyclic drugs have not been defined, but it is likely that inactivation of multiple targets has synergistic effects that irreversibly damage the target cells [49]. The mechanism of action of gold(I) compounds is not well understood but also involve adduct formation and likely inactivation of multiple targets [50]. The multiplicity of cellular targets for both drug classes can provide an explanation for their similar activities against *T*. *foetus* and *T*. *vaginalis*, since even differences in the sequence or structure of any particular target are unlikely to interfere with the overall effectiveness of these drugs. It must be noted, however, that while not statistically significant, *T*. *foetus* had generally lower pIC50s for each compound of the two drug classes in the *in vitro* screening assays, which was also observed in prior studies with fewer compounds [22, 40]. Another explanation for differential drug susceptibility could be differential membrane permeability between the two parasites [52], which may allow for increased passive diffusion of small molecules, including potentially the tested antimicrobial drugs whose molecular masses are <800 Da, and thus greater susceptibility of *T*. *vaginalis*.

Notably, we found little or no activity differences in the MLC assays, perhaps suggesting that the IC50 growth assays and MLC survival assays test slightly different drug properties or are differentially impacted by parasite characteristics not directly related to the drug action. For example, the IC50 assays could be affected by the faster growth rate of *T*. *foetus* over *T*. *vaginalis*. Drugs such as 5-nitroheterocyclic compounds that form covalent adducts may be consumed during the culture period, so a faster growing organism may deplete drugs more rapidly, thereby requiring apparently higher IC50 values to achieve 50% maximal growth inhibition. By comparison, MLC assays measure the ability of a drug to kill all target organisms, which typically requires higher drug levels, so the impact of drug consumption would be less apparent. Nonetheless, both IC50 and MLC provide valuable information for comparative assessment of the drug impact of *T*. *foetus* and *T*. *vaginalis*, and clearly show that the drug susceptibility of *T*. *foetus* closely resembles *T*. *vaginalis* in regard to 5-nitroheterocyclic and gold(I) compounds. Consequently, our demonstration of *in vivo* efficacy for one of the gold(I) compounds is promising for its ultimate *in vivo* efficacy against *T*. *vaginalis*.

Contrary to the 5-nitroheterocyclic and gold(I) compounds, we observed marked differences in the activity of proteasome inhibitors against *T*. *foetus* and *T*. *vaginalis*. Thus, *T*. *foetus* had significantly lower pIC50s for all tested drugs in this class compared to *T*. *vaginalis*. The target of these compounds is well defined and involves one or several of the three catalytic subunits of the proteasome [53, 54]. Differences in the sequence or fine structure of the relevant proteasome subunits between the two trichomonad species, which clearly exist based on our phylogenetic analysis, can probably impact the binding of specific inhibitors. In this context, it is important to note that the tested inhibitors were originally developed against the human proteasome [55] but not specifically selected for activity against either *T*. *vaginalis* or *T*. *foetus*. Screening of large proteasome inhibitor libraries may well identify compounds that are equally active against both trichomonads. It is therefore possible, that inhibitors could be found that are more active against *T*. *foetus* than *T*. *vaginalis* and even human cells, which could represent a promising starting point for new veterinary drugs against bovine tritrichomoniasis [56]. Nevertheless, based on the phylogenetic and functional analyses, we were able to conclude that no fundamental differences exist in the 20S proteasomes of *T*. *foetus* and *T*. *vaginalis*. We could also demonstrate that one proteasome inhibitor, bortezomib, has low micromolar or better activity against both *T*. *foetus* and *T*. *vaginalis* and can eliminate *T*. *foetus* infection *in vivo*, underlining that drug candidates with adequate activity against *T*. *foetus* can be active *in vivo* even if their relative potency in *T*. *foetus* does not closely mimic the findings in *T*. *vaginalis*.

While this study focused on female mice, it is also important to acknowledge that males are carriers of *T*. *vaginalis* and at times may suffer symptoms. Male animal models of *T*. *vaginalis* infection via urethral injection and anesthesia have been established in mice [47] and rats [57]. In future studies, it would be of interest to evaluate drug efficacy in these models for potential treatment of male trichomoniasis.

In summary, the *T*. *foetus* murine infection models described here have excellent utility as surrogate *in vivo* models for drug development campaigns for new drugs against *T*. *vaginalis*, particularly if multiple molecular drug targets are predicted. Caution should be exercised, however, for compounds with singular targets if those targets differ significantly between the two trichomonad species. In such situations, it would be advisable to conduct careful *in vitro* compound testing beforehand to confirm that drug activity is not significantly different between the two organisms or at last remains sufficiently high in *T*. *foetus* that *in vivo* efficacy testing is not likely to yield false negative results.

## Supporting information

S1 Table Activity of antimicrobial compounds against diverse T. foetus and T. vaginalis strains. *T*. *foetus* and *T*. *vaginalis* cultures were incubated for 24 hours with a range of drug concentrations, ATP content was assayed as a measure of cell growth and viability, and pIC50 values were calculated from the resulting concentration-response curves. Data are shown as mean ± SE of three or more independent experiments. IC50 values were derived from the mean pIC50. The highest tested drug concentration was 20 μM, so lack of growth inhibition at that concentration is listed as IC50 >20 μM.
(PDF)

S1 Raw images Raw blot and gel images. (PDF)
