# Supplementary material for: Vaginal Tritrichomonas foetus infection in mice as an in vivo model for drug development against Trichomonas vaginalis
Source: PLoS One. 2024 Oct 1;19(10):e0308672. doi: 10.1371/journal.pone.0308672 (PMC11444383; doi:10.1371/journal.pone.0308672)
Supplement: S1 Table — T. foetus and T. vaginalis cultures were incubated for 24 hours with a range of drug concentrations, ATP content was assayed as a measure of cell growth and viability, and pIC50 values were calculated from the resulting concentration-response curves. Data are shown as mean ± SE of three or more independent experiments. IC50 values were derived from the mean pIC50. The highest tested drug concentration was 20 μM, so lack of growth inhibition at that concentration is listed as IC50 >20 μM. (PDF) [file pone.0308672.s001.pdf]

Supplemental Table 1. Activity of antimicrobial compounds against diverse *T. foetus* and *T. vaginalis* strains

		T. foetus strains								*T. vaginalis* strains					
		D1		232		924		166		F1623		R88		S1489	
Drug class	Drug name	pIC50 (mean ± SE)	IC50 (µM)	pIC50 (mean ± SE)	IC50 (µM)	pIC50 (mean ± SE)	IC50 (µM)	pIC50 (mean ± SE)	IC50 (µM)	pIC50 (mean ± SE)	IC50 (µM)	pIC50 (mean ± SE)	IC50 (µM)	pIC50 (mean ± SE)	IC50 (µM)
5-nitro heterocyclic compounds	Metronidazole	5.8 ± 0.15	1.5	5.6 ± 0.03	2.4	5.7 ± 0.09	1.9	5.8 ± 0.04	1.8	6.2 ± 0.06	0.62	6.0 ± 0.05	1.0	6.6 ± 0.13	0.26
	Ronidazole	6.0 ± 0.07	1.0	5.4 ± 0.32	3.9	5.6 ± 0.07	2.5	5.7 ± 0.18	1.8	6.1 ± 0.17	0.87	5.9 ± 0.03	1.3	6.3 ± 0.02	0.46
	Tinidazole	5.2 ± 0.19	6.0	5.1 ± 0.11	7.9	5.1 ± 0.11	7.6	5.3 ± 0.11	4.6	5.6 ± 0.06	2.3	5.6 ± 0.14	2.9	5.9 ± 0.09	1.2
	Nitaxozanide	5.5 ± 0.07	2.9	5.2 ± 0.14	6.0	5.6 ± 0.12	2.4	5.4 ± 0.09	4.1	5.8 ± 0.04	1.6	5.5 ± 0.04	3.5	6.0 ± 0.01	0.96
	Nithiamide	5.6 ± 0.07	2.4	5.3 ± 0.17	4.8	5.4 ± 0.07	3.9	5.4 ± 0.11	3.7	6.1 ± 0.06	0.89	5.7 ± 0.11	2.1	6.5 ± 0.04	0.36
Gold (I) compounds	Auranofin	6.6 ± 0.03	0.27	6.5 ± 0.07	0.30	6.1 ± 0.23	0.80	6.1 ± 0.15	0.88	6.5 ± 0.08	0.29	6.8 ± 0.19	0.17	7.0 ± 0.23	0.11
	CPD4	6.6 ± 0.10	0.25	6.3 ± 0.04	0.54	6.4 ± 0.04	0.42	6.2 ± 0.20	0.71	6.7 ± 0.20	0.18	6.8 ± 0.08	0.17	6.6 ± 0.30	0.26
	CPD10	5.2 ± 0.16	7.0	5.4 ± 0.20	4.1	5.3 ± 0.17	5.5	4.9 ± 0.06	12	5.9 ± 0.05	1.3	6.2 ± 0.12	0.69	6.2 ± 0.17	0.64
	CPD11	5.8 ± 0.12	1.6	5.5 ± 0.29	3.2	5.7 ± 0.16	1.8	5.3 ± 0.18	5.2	5.9 ± 0.11	1.2	5.9 ± 0.21	1.3	6.8 ± 0.29	0.17
	CPD12	6.2 ± 0.30	0.59	5.9 ± 0.12	1.4	6.0 ± 0.13	0.90	5.7 ± 0.23	1.9	6.1 ± 0.09	0.75	5.9 ± 0.26	1.2	6.7 ± 0.44	0.19
	CPD14	6.4 ± 0.04	0.39	6.0 ± 0.04	1.1	6.1 ± 0.06	0.72	5.8 ± 0.13	1.6	6.4 ± 0.04	0.40	6.3 ± 0.17	0.49	6.6 ± 0.08	0.26
	CPD15	6.5 ± 0.14	0.30	6.4 ± 0.05	0.39	6.3 ± 0.11	0.55	5.8 ± 0.13	1.6	6.5 ± 0.05	0.30	6.5 ± 0.20	0.30	6.8 ± 0.04	0.16
	Myochrysine	<4.7	>20	4.7 ± 0.03	19	<4.7	>20	4.7 ± 0.01	19	<4.7	>20	<4.7	>20	<4.7	>20
Proteasome inhibitors	Bortezomib	6.1 ± 0.15	0.74	5.9 ± 0.23	1.2	5.7 ± 0.06	2.2	6.0 ± 0.16	1.0	6.6 ± 0.08	0.24	7.0 ± 0.12	0.11	6.7 ± 0.15	0.19
	Ixazomib	5.5 ± 0.17	3.3	5.3 ± 0.27	4.8	5.0 ± 0.07	9.9	5.5 ± 0.18	3.5	6.4 ± 0.11	0.36	6.8 ± 0.11	0.16	6.8 ± 0.09	0.17
	Carfilzomib	4.8 ± 0.05	17	4.8 ± 0.10	16	4.8 ± 0.05	18	4.8 ± 0.04	16	5.2 ± 0.03	5.8	5.8 ± 0.63	1.7	7.1 ± 0.17	0.09
Benzimidazole compounds	Lansoprazole	4.7 ± 0.01	20	<4.7	>20	4.7 ± 0.02	19	<4.7	>20	4.8 ± 0.05	16	<4.7	>20	<4.7	>20
	Omeprazole	4.8 ± 0.05	18	<4.7	>20	4.7 ± 0.03	19	<4.7	>20	<4.7	>20	4.7 ± 0.02	19	<4.7	>20
	Pantoprazole	<4.7	>20	<4.7	>20	<4.7	>20	<4.7	>20	<4.7	>20	4.7 ± 0.01	19	<4.7	>20
	Albendazole	4.7 ± 0.01	20	4.7 ± 0.01	20	4.7 ± 0.03	18	<4.7	>20	<4.7	>20	<4.7	>20	<4.7	>20
	Fenbendazole	<4.7	>20	<4.7	>20	4.7 ± 0.03	18	<4.7	>20	<4.7	>20	<4.7	>20	4.7 ± 0.02	19
Miscellaneous	Disulfiram	5.5 ± 0.10	3.4	5.1 ± 0.33	7.6	5.4 ± 0.15	4.2	5.4 ± 0.35	4.3	5.8 ± 0.25	1.5	5.4 ± 0.58	4.4	5.9 ± 0.58	1.4

Cultures of *T. foetus* and *T. vaginalis* were incubated for 24 hours with a range of concentrations of the indicated drugs. ATP content was determined as a measure of cell growth and viability, and pIC50 values were calculated from the concentration-responses curves. Data are shown as mean ± SE of three or more independent experiments. IC50 values were derived from the mean pIC50. Drugs with less than 50% inhibition at the highest tested concentration are shown as IC50 >20 µM and pIC50 <4.7.
