# Supplementary material for: Vaginal Tritrichomonas foetus infection in mice as an in vivo model for drug development against Trichomonas vaginalis
Source: PLoS One. 2024 Oct 1;19(10):e0308672. doi: 10.1371/journal.pone.0308672 (PMC11444383; doi:10.1371/journal.pone.0308672)
Supplement: S1 Raw images — (PDF) [file pone.0308672.s002.pdf]

Supporting Information File 1

Vaginal Tritrichomonas foetus infection in mice as an in vivo model for drug development against Trichomonas vaginalis

Noelle M. Nieskens, Yukiko Miyamoto, Brianna M. Hurysz,
Anthony J. O'Donoghue, and Lars Eckmann

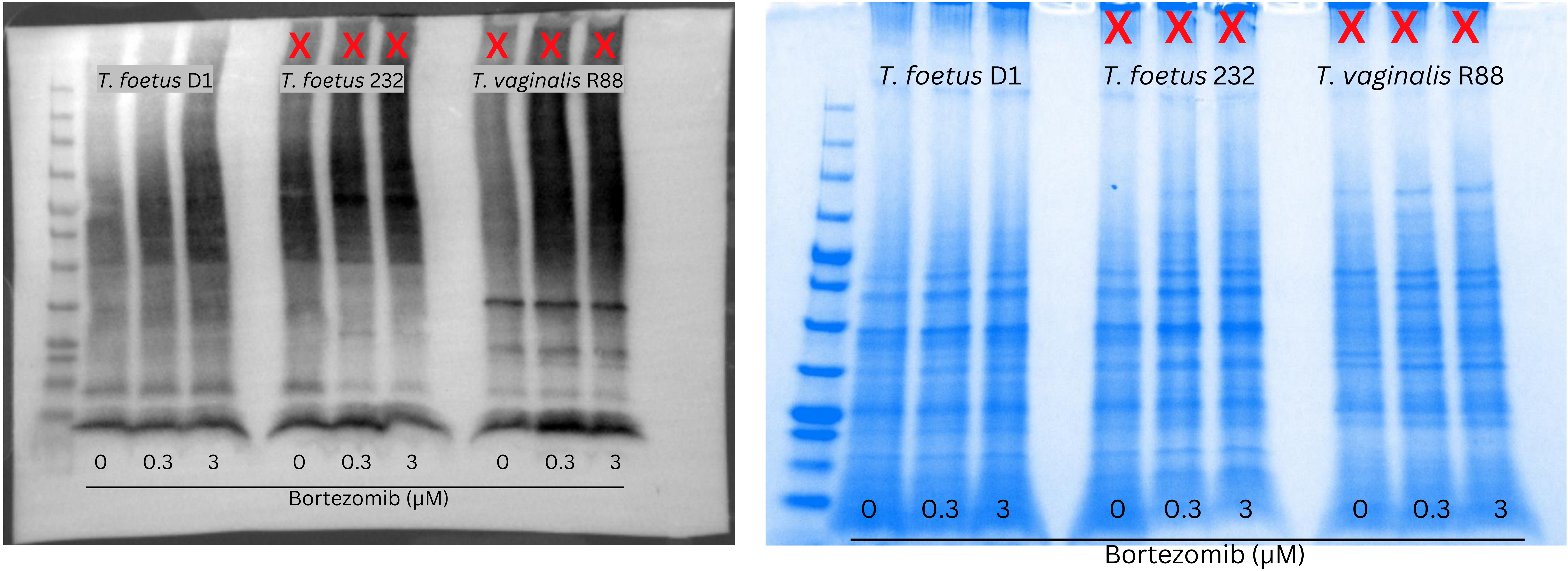

Fig. 7A

Image taken with ChemiDoc XRS+ (Bio-Rad)

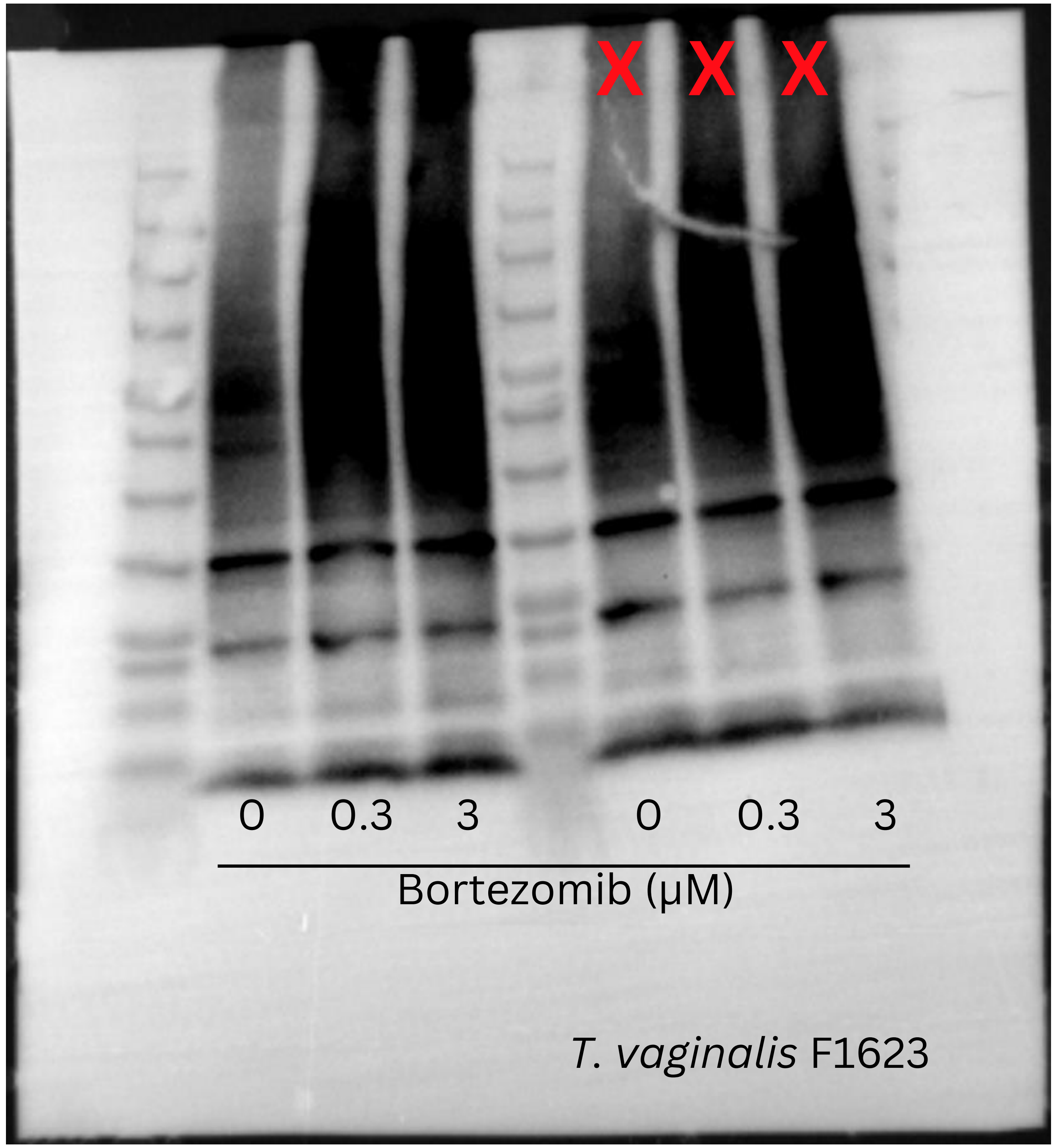

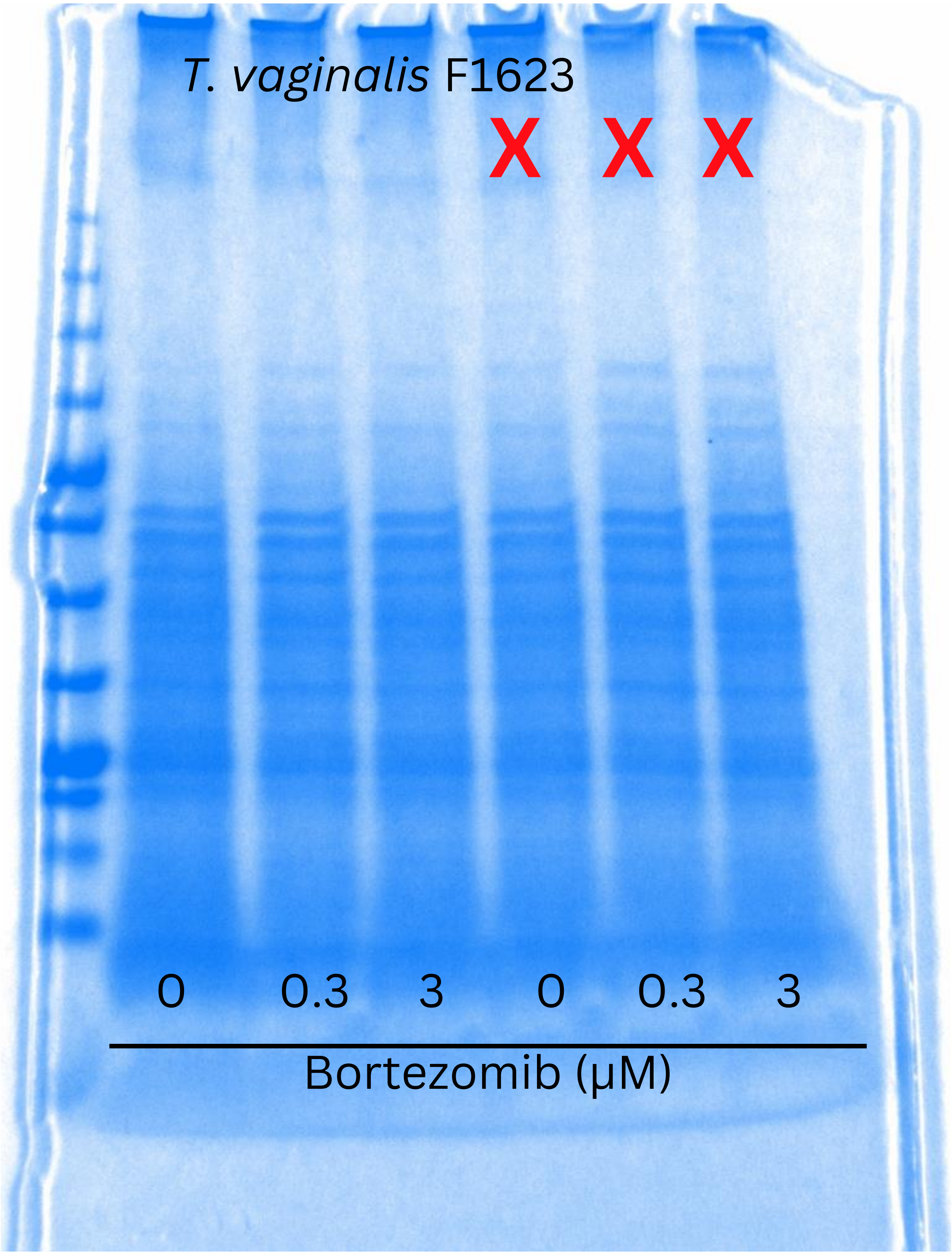

Fig. 7A

Image taken with ChemiDoc XRS+ (Bio-Rad)

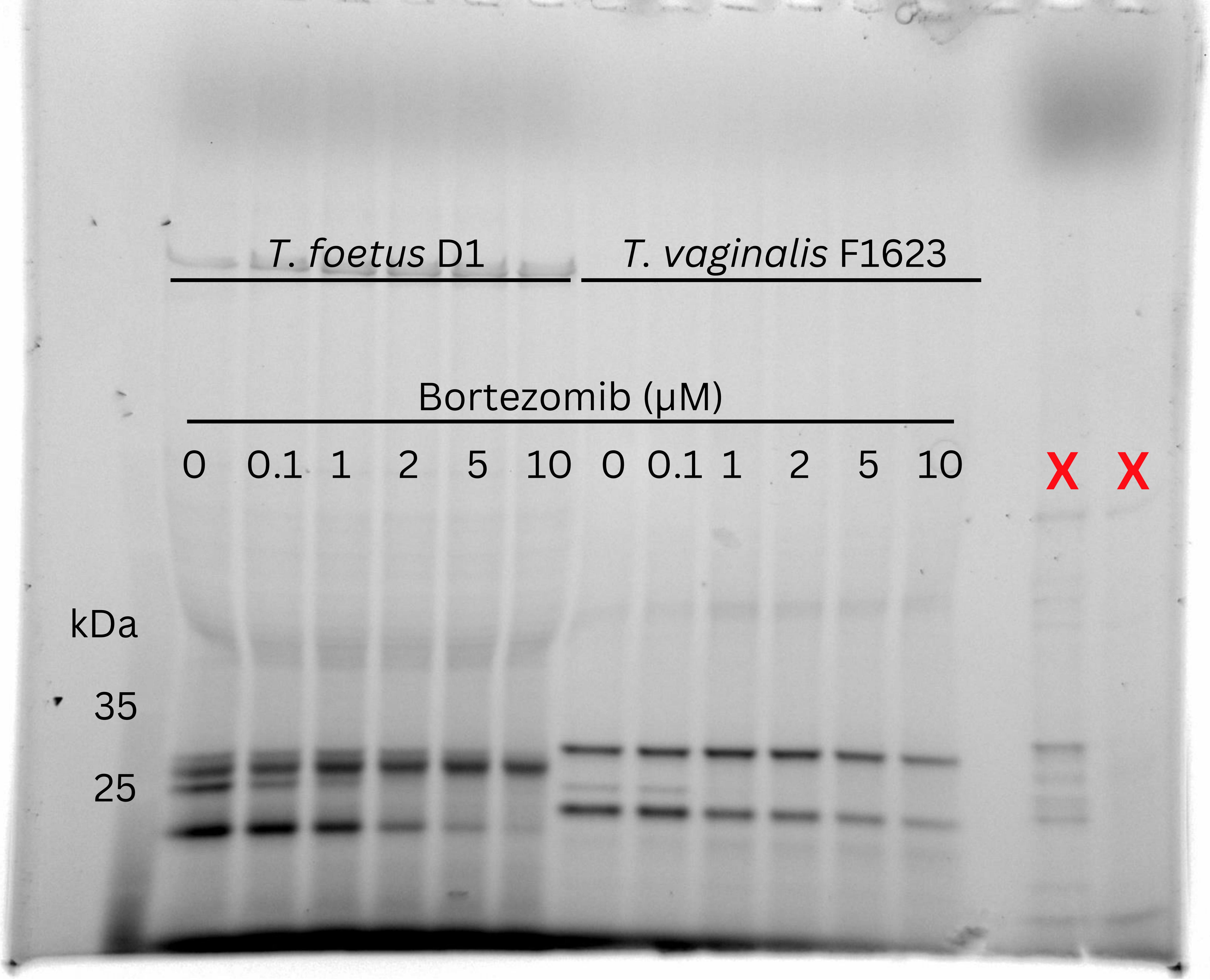

Fig. 7B
Image taken with ChemiDoc XRS+ (Bio-Rad)
